# DNA methylation and epigenomics: new technologies and emerging concepts

**DOI:** 10.1186/s13059-015-0674-5

**Published:** 2015-05-21

**Authors:** Aniruddha Chatterjee, Michael R Eccles

**Affiliations:** Department of Pathology, Dunedin School of Medicine, University of Otago, 270 Great King Street, Dunedin, 9054 New Zealand; Maurice Wilkins Centre for Molecular Biodiscovery, Level 2, 3A Symonds Street, Auckland, New Zealand

## Abstract

A report of the Keystone Symposia joint meetings on DNA Methylation and Epigenomics held in Keystone, Colorado, USA, 29 March to 3 April, 2015.

## Introduction

This year, the Keystone Symposia hosted concurrent meetings on DNA methylation and epigenomics. Multiple sessions were jointly held between the two meetings, and in total the number of participants at both meetings was one of the largest ever at Keystone. A notable aspect of the two meetings was the relatively large number of new and increasingly powerful epigenetic technologies that have been developed recently, ranging from novel single-cell epigenetic profiling to ligation-free Hi-C (Table 1). Many new findings and novel concepts were also discussed at the meeting, particularly around the epigenetics of differentiation and development, as well as disease, pluripotency and stem cells, to name just a few. The meeting opened with a Keynote Address by Adrian Bird (University of Edinburgh, UK), who reported on altered DNA methylation regions in cancer, and brain genomes that coincide with regions of altered base composition. AT-rich DNA regions are relatively less methylated, while CG-rich regions are relatively more methylated, leading to speculation that base composition in the genome impacts the methylome. The proteins bound to DNA are known to influence DNA methylation, and these proteins are in turn influenced by DNA base composition.

**Table 1:** Recent technological developments in epigenetics reported at the joint meetings

Technique	Description	Speaker
Single-cell DNA methylation	NA	Wolf Reik (Babraham Institute, UK)
Single-cell Hi-C	NA	Peter Fraser (Babraham Institute, UK)
Ligation-free Hi-C	Ligation-free assay for genome-wide chromatin interactions	Robert Beagrie (MRC Clinical Sciences Centre, UK)
NOMe-seq	Assay developed in combination with ChIP-seq to analyze cancer in patients treated with DNMT inhibitors	Peter Jones (Van Andel Research Institute, USA)
RAP	Method to identify genome-wide localization of a lncRNA	Mitchell Guttman (California Institute of Technology, USA)
RAP-MS	Method to identify lncRNA-protein interactions	Mitchell Guttman
SNP-FISH	Technique developed by Arjun Raj and colleagues to enable allele-specific expression analysis of single cells, or small groups of cells	Marisa Bartolomei (University of Pennsylvania Perelman School of Medicine, USA)
HaploSeq	Haplotype reconstruction sequencing. *In situ* ligation to reconstruct long haplotype blocks	Bing Ren (University of California, San Diego, USA)
NET-seq	Approach that exploits the extraordinary stability of the DNA-RNA-RNA polymerase ternary complex to capture nascent transcripts directly from live cells	L. Stirling Churchman (Harvard Medical School, USA)
Methylation analysis software for the DRAGEN Bio-IT processor	Software used to carry out methylation analysis of a genome in 17 minutes	Joseph Ecker (HHMI/The Salk Institute for Biological Studies, USA)

*ChIP* chromatin immunoprecipitation; *DNMT* DNA methyltransferase; *HaploSeq* haplotype reconstruction using proximity-ligation and shotgun sequencing; *Hi-C* comprehensive detection of chromatin interactions in the mammalian nucleus; *IT* information technology; *lncRNA* long non-coding RNA; *NA* ; *NET-seq* native elongating transcript sequencing; *NOMe-seq* nucleosome occupancy and methylome sequencing; *RAP* RNA antisense purification; *RAP-MS* RNA antisense purification with mass spectrometry; *SNP-FISH* single nucleotide polymorphism and fluorescence *in situ* hybridization

## DNA methylation, differentiation and chromatin

Key questions in epigenetics are what drives cell differentiation, and what is the role in this process of the establishment of chromatin marks or DNA methylation? Related to this, how are the patterns of chromatin or DNA methylation initially primed, and how does genetic or epigenetic variation impact development? A number of talks focused on these questions. First, Alexander Meissner (Harvard University, Broad Institute, USA) discussed targeted DNA methylation as embryonic stem cells (ESCs) differentiate into the endoderm. Knockout (KO) of DNA (cytosine-5)-methyltransferase 3A (DNMT3A) in differentiating ESCs leads to a lack of methylation acquired *de novo* at many sites, including genes such as *Nanog* and *Foxa2*. Nonetheless, the cells are still able to form teratomas when they are injected into mice. Knockout of both DNMT3A and DNMT3B causes passage-dependent loss of DNA methylation, albeit at very slow rates. Interestingly, deletion of DNA (cytosine-5)-methyltransferase 1 (DNMT1) causes cell death in ESCs. Ryan Lister (University of Western Australia, Australia) addressed whether *de novo* methylation occurring in gene promoters is (solely) responsible for gene silencing. He described epigenetic manipulation in cells by inducing the expression of a zinc finger-DNMT3A fusion protein, resulting in high levels of widespread, *de novo* methylation of gene promoters. Strikingly, gains in promoter DNA methylation simultaneously coexist with active histone marks on many gene promoters, and are insufficient to transcriptionally repress most genes. Removal of zinc finger-DNMT3A overexpression leads to a rapid return to an unmethylated state. Bing Ren (University of California, San Diego, USA) described the creation of a novel, chromosome-spanning haplotype reconstruction strategy (HaploSeq; Table 1), which revealed extensive allelic biases and considerable variation in both chromatin state and transcription from identical human tissues in different individuals. Allelic differences of chromatin state involve *cis*-regulatory elements and are associated with allelic differences in transcription factor binding due to local sequence variations.

## Reading, writing and erasing epigenetic marks: retrotransposons, regulators and remodeling

The establishment (writing), recognition (reading) and erasing of epigenetic marks in the cell are important features central to the reversibility of epigenetic modifications. Retrotransposons in the genome, for example, are usually heavily methylated and silenced; however, on some occasions it appears that they are also expressed, as discussed further on. Furthermore, the folding of chromosomes into active or inactive domains is at least in part a phenomenon that is epigenetically regulated. Elphège P Nora (Gladstone Institute, USA), from the laboratory of Benoit Bruneau, engineered mouse ESCs that are completely depleted of transcriptional repressor CTCF. Using carbon-copy chromatin conformation capture (5-C) at the *Xic* locus, they showed that the folding of chromosomes into topologically associating domains (TADs) is dependent on CTCF. From this work they concluded that: (1) CTCF is not essential for short-term ESC survival; (2) chromosome conformation is continuously driven by the action of CTCF; (3) folding into TADs is not an intrinsic property of chromosomes; and (4) that histone H3 trimethyl Lys27 domains in ESCs are not constrained by CTCF. Joseph Costello (University of California, San Francisco, USA) reported the widespread existence of intermediate DNA methylation domains in eight human cell types from four different tissues. He reported that intermediate DNA methylation domains are remarkably consistent across individuals, and conserved between humans and mice. Intermediate DNA methylation domains have an average length of 300 bp, and 70 % of them are independent of allelic status (also known as patchy methylation). The regions containing intermediate DNA methylation domains also show intermediate levels of gene transcription and exon usage. Sriharsa Pradhan (New England Biolabs, USA) reported on writing, reading and erasing functions associated with the amino acid Lys142 of DNMT1. The writer enzyme histone-lysine *N*-methyltransferase SETD7 induces monomethylation of Lys142, leading to proteosomal degradation of DNMT1. Surprisingly, DNMT1 is also found to interact specifically with several small inhibitory RNAs, which bind to the active (catalytic) site of DNMT1, competitively inhibiting its methylation. Overexpression of exogenous miRNAs, such as miR-155, leads to aberrant genomic DNA methylation, resulting in hypomethylation of certain genomic regions. Joanna Wysocka (Stanford University School of Medicine, USA) spoke about the human endogenous retrovirus group K (HERV-K) family of transposable elements, which retain open reading frames and are usually hypermethylated in human somatic cells. DNA hypomethylation and octamer-binding transcription factor 4 expression in the human embryo acted synergistically to promote the expression of HERV-K full-length transcripts, and virus-like particles in the blastocyst were detectable by electron microscopy. Antiviral restriction factors are upregulated and may act to protect the developing embryo from infection by exogenous viruses and active endogenous retroviruses.

Several speakers highlighted new techniques in this area of research. L Stirling Churchman (Harvard Medical School, USA) reported that RNA polymerase is a uniquely sensitive ‘reader’ of sequence features and epigenomic states. Her lab recently developed a new technique called native elongating transcripts sequencing (NET-seq; Table 1), which maps active RNA polymerase II *in vivo*. Using this technique, widespread divergent antisense transcription is detected in 77 % of all expressed genes in human cells, and convergent transcription, a characteristic of genes that are expressed at very low levels, in 25 % of promoters. Using NET-seq, RNA polymerase can detect exons and splicing fates, while pronounced pausing is observed at retained and constitutive exons, but not at alternatively spliced exons. Using a new low-input chromatin immunoprecipitation sequencing (ChIP-seq) method, Matthew Lorincz (University of British Columbia, Canada) reported that class I and class II long terminal repeat (LTR) retrotransposons are marked with histone H3 lysine 9 trimethylation (H3K9me3) in embryonic day 13.5 primordial germ cells, the stage in gametogenesis when DNA methylation levels are at their lowest. As in the preimplantation embryo, however, the H3K9me3-marked LTR elements are resistant to DNA demethylation. By using a germ line-specific conditional KO of the H3K9 histone-lysine *N*-methyltransferase SETDB1, their observations suggest that the H3K9me3 mark may promote the persistence of DNA methylation during the developmental stages, when maintaining the DNA methylation machinery is tightly regulated.

## Interrelated roles of non-coding RNAs and chromatin modification

There are over 10,000 long non-coding RNAs (lncRNAs) in humans and mice, with many affecting gene expression. Several talks in this session addressed the major questions surrounding lncRNA activity related to chromatin modifications. One of the standout talks in this group was given by Mitchell Guttman (California Institute of Technology, USA), who reported on his lab’s studies of *Xist* lncRNA localization, spreading and silencing across the X chromosome. Continuing the theme of emerging technology, the Guttman lab developed a novel method called RNA antisense purification (RAP; Table 1), which allows genome-wide localization of a lncRNA, and applied this method to study the localization of the *Xist* lncRNA in a doxycycline-inducible cell culture system. They were able to determine that *Xist* localizes to initial target sites based on the three-dimensional structure of the X chromosome. By adapting the RAP method to enable comprehensive detection of the proteins that associate with *Xist*, Guttman showed that silencing the X chromosome requires a set of proteins, including a large scaffold protein called SHARP, which silences transcription by interacting with histone deacetylase 3 (HDAC3) to evict RNA polymerase II from chromatin. Overall, their very comprehensive studies suggest a model whereby *Xist* and other lncRNAs may act as regulatory scaffolds to localize chromatin and regulate transcription at target sites.

Other talks in these sessions focused on the activity of small non-coding RNAs. Natasha Weiser (University of Michigan, USA) presented work in *Caenorhabditis elegans*, where MORC-1 protein regulates the balance of active and silent chromatin by binding euchromatin, promoting the removal of activation marks such as H3K4me3, and allowing the deposition of repressive H3K9me3 marks by the nuclear RNA interference (RNAi) pathway. Loss of critical components of the RNAi pathway lead to the transgenerational loss of H3K9me3 at target genes.

## Genomic imprinting

Another significant session of the conference focused on emerging research in genomic imprinting, the epigenetic mechanism by which certain genes are expressed in a parent-of-origin-specific manner. Anne Ferguson-Smith (University of Cambridge, UK) reported that the maintenance of methylation at imprinting control regions (ICRs) during the widespread demethylation that occurs in the zygotic preimplantation embryo occurs via the binding of the Krüppel-associated box (KRAB)-zinc finger protein 57 (ZFP57) to methylated alleles of ICRs. Maternal-zygotic mutation of the *Zfp57* gene (that is, homozygous embryos derived from homozygous eggs) leads to rapid loss of imprints during zygotic reprogramming.

## Epigenetics of disease, epigenetic biomarkers and therapeutics

Epigenetic alterations occur very frequently in cancer, and epigenetic marks can also be exploited for their prognostic or diagnostic value as biomarkers. Peter Jones (Van Andel Research Institute, USA) used a new nucleosome occupancy and methylome sequencing assay (NOMe-seq; Table 1) developed in combination with ChIP-seq to analyze cancers in patients treated with DNMT inhibitors, revealing reorganization of nucleosome positioning-phasing accompanying DNA methylation loss in CpG island (CGI) promoters, in association with gene activation. Interestingly, phasing around CTCF binding sites remains largely unaffected in response to DNA demethylation events in cancer. Peter Laird (Van Andel Research Institute, USA) updated research to define the CGI methylator phenotype (CIMP) from analyses involving approximately 5,000 cancer specimens spanning 12 cancer types. Genes previously not known to be epigenetically silenced were identified, as well as being integrated with other levels of information (such as the presence of common DNA mutations) to enhance the value of CIMPs. He also described a mouse model where engineered mice with an inducible repression of DNMT1 were crossed with APC^min^ mice, which suppressed intestinal polyp formation through transcriptional regulation of DNMT1. Stephan Beck (University College London, UK) presented data on epigenetic biomarkers for two different conditions. The first was a predictive biomarker for severe acute graft-versus-host disease (GVHD) in healthy donors prior to haematopoietic stem cell transplantation. This marker has been validated in the lab and a large-scale study is now under way. The second was a predictive biomarker for differentiation capacity following reprogramming of adult cells into induced pluripotent stem cells (iPSCs) using Yamanaka factors and differentiating iPSCs into the endoderm lineage. Surprisingly, Beck’s team found that non-CpG methylation patterns are a better biomarker for this than traditional CpG methylation.

## Pluripotency and stem cells

Epigenetics is a key area of study in the fields of pluripotency and stem cell research, and several interesting talks in this session addressed ongoing experiments related to how epigenetic marks are dynamic and functional throughout the process of stem cell differentiation. Wolf Reik (Babraham Institute, UK) outlined evidence that ESCs undergo epigenetic priming by *de novo* methylation as they transit out of naive pluripotency. Based on the fact that these cells express high levels of DNMT3 and ten-eleven translocation enzymes, he proposed that continuous reprogramming might create epigenetic heterogeneity in the cell population. Single-cell DNA methylation analysis revealed that enhancers (marked by H3K4me1) and low CpG content promoters are the most variably methylated genomic elements in ESCs. Variable methylation in regulatory elements is associated with variable transcription as assessed by single-cell RNA sequencing. They are now addressing the question whether a similar system operates *in vivo* in the embryo during gastrulation. Bradley Cairns (HHMI/University of Utah, USA) reported that in spermatogonial stem cells and sperm, the promoters or enhancers of *Nanog*, *Prdm14* and *Sox2*, which are silent in spermatogonial stem cells and adult germ line stem cells, bear particular chromatin and DNA methylation attributes that may ‘poise’ them for reactivation after fertilization, and which may underlie their spontaneous ability to form ES-like cells *in vitro*. They also observed this ‘poising or bivalency’ at the promoters of many embryonic transcription factors in the germ line, which may enable their expression in embryos.

Several other talks focused on how epigenetic marks may be altered in disease. Margaret Goodell (Baylor College of Medicine, USA) discussed epigenetic development of the haemopoietic system. DNMT3A mutations are associated with approximately 20 % of certain types of haematological malignancies. DNMT3A conditional KO mice carry out haematopoiesis at a greater rate than wild-type mice, whereas DNMT3B KO diminishes blood production. Haemopoietic differentiation in double KOs is almost completely blocked, mediated in part by stabilized beta-catenin protein. Mice transplanted with DNMT3A KO haemopoietic stem cells all develop haematological malignancies within approximately 400 days, with pathological characteristics similar to haematological malignancies in patients with DNMT3A mutations. Jeanne Loring (The Scripps Research Institute, USA) described reprogramming of iPSCs derived from fragile X syndrome patients to develop a therapy for these patients. Cells carrying a fragile X mutation have DNA methylation at the fragile X mental retardation 1 (*Fmr1*) locus, but due to somatic mosaicism it is possible to identify cells with expanded repeats that are unmethylated at the *Fmr1* gene; *Fmr1* methylation-negative iPSCs differentiate into neurons with a slight developmental delay compared to wild-type iPSCs. Some genes, including autism-associated genes, are more frequently affected in neuronally differentiated iPSCs from fragile X syndrome patients.

## Concluding remarks

This meeting covered many new developments and concepts in the field of DNA methylation and epigenomics. However, due to the large number of extremely interesting and high-quality talks, as well as posters presented at the meeting, this report only gives a brief glimpse of the whole meeting, and unfortunately we could not include many other excellent presentations. In addition, we have reported a number of new and very exciting technological developments (Table 1); as a result, it is very likely that there will be much exciting research occurring in this field in the very near future.

## Abbreviations

5-C: carbon-copy chromatin conformation capture
bp: base pair
CGI: CpG island
ChIP: chromatin immunoprecipitation
ChIP-seq: chromatin immunoprecipitation sequencing
CIMP: CpG island methylator phenotype
DNMT: DNA methyltransferase
DNMT1K142: DNA methyltransferase 1 lysine 142
DNMT3A: DNA (cytosine-5)-methyltransferase 3A
ESC: embryonic stem cell
*Fmr1*: fragile X mental retardation 1
GVHD: graft-versus-host disease
H3K9: histone H3 lysine 9
H3K9me3: histone H3 lysine 9 trimethylation
HaploSeq: haplotype reconstruction using proximity-ligation and shotgun sequencing
HDAC3: histone deacetylase 3
HERV-K: human endogenous retrovirus group K
Hi-C: comprehensive detection of chromatin interactions in the mammalian nucleus
ICR: imprinting control region
iPSC: induced pluripotent stem cell
IT: information technology
KO: knock out
KRAB: Krüppel-associated box
lncRNA: long non-coding RNA
LTR: long terminal repeat
miRNA: microRNA
NET-seq: native elongating transcript sequencing
NOMe-seq: Nucleosome occupancy and methylome sequencing
RAP: RNA antisense purification
RNAi: RNA interference
TAD: topologically associating domain
ZFP57: zinc finger protein 57

